
JOURNAL INFORMATION
==============================
NLM Title Abbreviation: Atlanta Med Surg J
NLM Journal ID: 0001010

Atlanta Medical and Surgical Journal

Publisher: Biomedical Journal Digitization Project

ARTICLE INFORMATION
==============================
PMCID: PMC8846911
PMID: 35825404
Article version: 1
Subjects: Editorial and Miscellaneous

Veratrum Viride in Dysmenorrhæa

Print publication date: 1857 Aug
Volume: 2
Issue: 12
First page: 767
Last page: 767
License: This work is free of known copyright restrictions. For more information, please see PMC Back Issue Digitization.
License URL: https://creativecommons.org/publicdomain/mark/1.0/

==============================

*Veratrum Viride in Dysmenorrhœa.*—Dr. Gorham recommends in the Medical Independent, the use of Veratrum Viride. He begins two days before the expected period, and gives three drops of the tinct. every three hours, with directions to increase one drop at each succeeding dose, until nausea comes on, and then to reduce the dose again to three or four drops. In one case the nausea with retching was produced by the seven drop dose. The next case was vomited freely by the six drop dose; and the third sickened and vomited at the two drop dose. The frequency of the pulse was much reduced in all cases, and menstruation in each case came on at a later period by three or four days than the patient expected, but without the least pains, the relief appearing to be complete.

*Memphis Medical Recorder.*

---

### SUBSCRIPTIONS RECEIVED.

Drs. W. E. Wilson, Ill., 1st and 2d vols.; C. R Moore, Ga., 2d vol.; John Rambo, Ga., 1st and 2d vols.; S. C. Hitchcock, Ga., 2d vol.; H. H. Johnson, Ga., 1st vol.; Wm. Booth, Fla., 2d and 3d vols.; J. J. Scott, Miss., 2d vol.; T. T. Mounger, Fla., 2d vol.; Dean & Dupree, Ga., 1st and 2d vols.; N. B. Drewry, Ga., 2d vol.; J. C. Story, Ala., 2d vol.; J. W. Kinnebrew, Ga., 1st vol.; John Rhea, Ga., 2d vol.; Wm. McLean, Ga., 2d vol.; S. H. Freeman, Ga., 2d vol.; Wm. Booth, Fla., 1st and 2d vols.; J. B. Forbes, Ga., 1st and 2d vols.

---

### UNIVERSITY OF NASHVILLE.

MEDICAL DEPARTMENT.—SESSION 1857-8.—The Seventh Annual Course of Lectures in this Institution will commence on Monday, the 3d of November next, and continue till the first of the ensuing March.

THOMAS R. JENNINGS, M. D., Professor of Anatomy.

J. BERRIEN LINDSLEY, M. D., Professor of Chemistry and Pharmacy.

C. K. WINSTON, M. D., Professor of Materia Medica and Medical Jurisprudence.

A. H. BUCHANAN, M. D., Professor of Surgical Anatomy and Physiology.

JOHN M. WATSON, M. D., Professor of Obstetrics and the Diseases of Women and Children.

PAUL F. EVE, M. D., Professor of Principles and Practice of Surgery.

W. K. BOWLING, M. D, Professor of Institutes and Practice of Medicine.

WILLIAM T. BRIGGS. M. D., Adjunct Professor and Demonstrator of Anatomy.

The Anatomical Rooms will be opened for students on the first Monday in October, [the 5th].

A *Preliminary Course* of Lectures, free to all Students, will be gfven by the Professors, commenceing also on the first Monday of October.

The Tennessee State Hospital under the direction of the Faculty is open to the Class free of charge.

A Clinique has been established in connection with the University, at which operations are performed and cases prescribed for and lectured upon in the presence of the class.

Amount of Fees for Lectures is $105; Matriculation fee (paid once only), $5; Practical Anatomy, $10; Graduation Fee, $25.

Good boarding can be procured for $3 to $4 per week, For surther information or Catalogue apply to

PAUL F. EVE, M. D.,
*Dean of the Faculty.*

NASHVILLE, Tenn., July 16, 1857.
